# Antimicrobial and anti-inflammatory effects of *Eugenia brejoensis* essential oil in mice wounds infected by *Staphylococcus aureus*

**DOI:** 10.3389/fphar.2022.999131

**Published:** 2022-10-14

**Authors:** Roseana Muniz Diniz, Tatiany Gomes Ferreira Fernandes, Juliana Silva Pereira Mendonça, Lucas dos Santos Silva, Warlison Felipe de Silva Saminez, Patrícia Vieira de Oliveira, Erika Alves Da Fonseca Amorim, Cristiane Santos Silva e Silva Figueiredo, Clovis Macêdo Bezerra Filho, Maria Tereza dos Santos Correia, Márcia Vanusa da Silva, Joicy Cortez de Sá Sousa, Adrielle Zagmignan, Luís Cláudio Nascimento da Silva

**Affiliations:** ^1^ Laboratório de Patogenicidade Microbiana, Universidade Ceuma, São Luís, MA, Brazil; ^2^ Programa de Pós-graduação em Biologia Microbiana, Universidade Ceuma, São Luís, MA, Brazil; ^3^ Programa de Pós-graduação em Odontologia, Universidade Ceuma, São Luís, MA, Brazil; ^4^ Laboratório de Bioquímica de Proteínas, Centro de Biociências, Departamento de Bioquímica, Universidade Federal de Pernambuco, Recife, Brazil

**Keywords:** caatinga plants, volatile compounds, infectious diseases, skin healing, host-pathogen interactions, inflammatory mediators

## Abstract

*Eugenia brejoensis* Mazine (Myrtaceae) is source of an essential oil (EbEO) with anti-infective activities against *Staphylococcus aureus*. This study evaluated the antimicrobial and anti-inflammatory potentials of EbEO in *S. aureus*-infected skin wounds. The excisional lesions (64 mm^2^) were induced on Swiss mice back (6 to 8-week-old) that were allocated into 3 groups (*n = 12*): 1) non-infected wounds (CON); 2) wounds infected with *S. aureus* ATCC 6538 (Sa); 3) *S. aureus*-infected wounds and treated with EbEO (Sa + EbEO). The infected groups received approximately 10^4^ CFU/wound. The animals were treated with EbEO (10 µg/wound/day) or vehicle from the 1-day post-infection (dpi) until the 10th dpi. The clinical parameters (wound area, presence of exudate, edema intensity, etc.) were daily analyzed. The levels of inflammatory mediators (cytokines, nitric oxide, VEGF) and bacterial load were measured at the cutaneous tissue at 4th dpi and 10th dpi. Topical application of EbEO accelerated wound contraction with an average contraction of 83.48 ± 11.27 % of the lesion area until 6th dpi. In this period, the rates of lesion contraction were 54.28 ± 5.57% and 34.5 ± 2.67% for CON and Sa groups, respectively. The positive effects of EbEO on wound contraction were associated with significantly (*p* < 0.05) reduction on bacterial load and the release of inflammatory mediators (IL-6, IL-17A, TNF-α, NO and VEGF). Taken together, these data confirm the antimicrobial potential of EbEO and provide insights into its anti-inflammatory effects, making this essential oil an interesting candidate for the development of new therapeutic alternatives for infected cutaneous wounds.

## Introduction

*Staphylococcus aureus* is commonly found in the skin microbiota, making it a frequent contaminant of skin lesions (Parlet et al., 2019; Cheung et al., 2021). The presence of *S. aureus* in the wound may impair the healing process due to the prolongation of the inflammatory phase, characterized by the recruitment of leukocytes and release of cytokines (TNF-α, IFN-γ and IL-1β), nitric oxide (NO) and other effector molecules (Carvalho et al., 2018; Carneiro et al., 2021; Hatlen and Miller, 2021).

Among the plant-derived products, Essential oils (OE) are highlighted due their chemical diversity and several pharmacological properties including antioxidant, anti-inflammatory, antinociceptive, antimicrobial and healing action) (Bezerra Filho et al., 2020; Bunse et al., 2022; Costa et al., 2020a; Costa et al., 2020b; Ćavar Zeljković et al., 2022; Nascimento et al., 2022). These characteristics make the EO interesting alternative agents for the treatment of infected wounds (Nascimento et al., 2022; Omarizadeh et al., 2021).

*Eugenia brejoensis* Mazine (Myrtaceae) is an EO-bearing plant found in the Caatinga domain in northeastern Brazil. The essential oil obtained from its leaves (named as EbEO) has been characterized in previous studies as mainly composed of δ-cadinene, β-caryophyllene, α-muurolol, α-cadinol and bicyclogermacrene, even though the abundance of these phytoconstituents presented some differences accordingly to the study conditions probably due to seasonal changes in climate and soil composition and also the equipment used in the chemical characterization (Silva et al., 2015; Souza et al., 2017; Mendes et al., 2018). EbEO has broad spectrum antibacterial action and anti-*Trypanossoma cruzi* effect, besides the cytotoxicity towards HeLa carcinogenic cells (Bezerra Filho et al., 2020; Mendes et al., 2018; Silva et al., 2015; Santana et al., 2020). In particular, EbEO showed *in vivo* antimicrobial activity against *S. aureus* using larvae of *Galleria mellonella* and *Caenorhabditis elegans* as models (Bezerra Filho et al., 2020).

Despite these insights provided by these invertebrate models, the antimicrobial action of EbEO has not been reported in mammalian models. Thus, the present study aimed to evaluate the antimicrobial activity of the essential oil of EbEO in a murine model of skin infection caused by *S. aureus*.

## Material and methods

### Obtaining the essential oil of *E. brejoensis*

The plant material was obtained at Parque Nacional do Catimbau (Buíque, Brazil), processed and deposited in the Herbarium of Instituto Agronomico de Pernambuco (voucher number: IPA 84.033). EbEO was obtained from dried leaves by hydrodistillation for 4 h in a Clevenger-type apparatus. After extraction, the obtained EbEO was dried over anhydrous sodium sulfate and conserved in inert glass tubes protected from light. The sample of EbEO used in this study was characterized by Mendes et al. (2018) and has β-(E)-Caryophyllene (31.0%), δ-cadinene (20.0%), and bicyclogermacrene (12.0%) as major compounds (Mendes et al., 2018).

### Animals

The *in vivo* study was carried out at the animal facility of *Universidade CEUMA* (São Luís, Brazil), after approval by its institutional Animal Ethics Committee (Protocol No. 00013/18). Healthy Swiss mice (*n = 36*; 6–8 weeks of age) were kept in an airy room with an average temperature of 21°C, 12 h light-dark cycle and received water and food *ad libitum*. The experiments were performed in polypropylene cages allocated in ventilated rack with independent insufflation and exhaust systems were used to decrease the risk of infections.

### Infection of excisional wound with *S. aureus*

The mice were intramuscularly anesthetized (1 mg/kg xylazine hydrochloride and 50 mg/kg ketamine chloride). After, the dorsal region was trichotomized and cleaned with sterile 150 mM NaCl and 70% ethyl alcohol. The skin was demarcated using a sterilized adhesive paper mold (64 mm^2^) and the skin was removed with blunt-tipped scissors and dissection forceps. *S. aureus* ATCC 6538 (approximately 4.0 × 10^6^ CFU/wound) was added to each excisional lesion, except for CON Group which received 80 μL of saline solution (Carneiro et al., 2021).

### Experimental groups and topical treatment

The topical treatment started 1 day-post infection (dpi). The animals were distributed in three groups:

• CON (*n = 12*): animals with noninfected wounds and daily treated with sterile 2% DMSO (dimethyl sulfoxide).
• Sa (*n = 12*): Animals with infected wounds and daily treated with sterile 2% DMSO (dimethyl sulfoxide) solution.
• Sa + EbEO (*n = 12*): Animals with infected wounds and daily treated with 50 µL of 200 μg/ml EbEO solution (10 μg/wound). The essential oil solution (200 μg/ml) was dissolved in sterile 2% DMSO solution.

The treatment was performed during 10 days within a laminar flow to avoid external contamination. After the macroscopic examination, a cover was added to each lesion. The mice weight and temperature were also daily checked. The animals were euthanized by anesthetic overdose at 3rd or 10th dpi (6 animals/group each day). Tissue samples were collected for histological analysis, quantification of bacterial load and measurement of inflammatory mediators.

### Macroscopic evaluation of the lesion

The daily macroscopic evaluation was performed to calculate an index of severity (Ferro et al., 2019) based on the following parameters: wound area (0–7), amount of exudate (0–3), type of exudate (0–4), edema intensity (0–3), color of surrounding skin tissue (0–4), type of debridement tissue (0–3). All wounds were photographed and their area were calculated by equation: Wound Area (mm^2^) = π. R. r. Where “R” is the largest radius and “r” is the smallest radius. The degree of contraction was expressed as a percentage (%) and defined by the formula: 100 × (W_o_–W_i_)/W_o_. Where, W_o_ is the initial wound area and W_i_ is wound area on the respective day.

### Quantification of bacteria in wounded tissue

The dorsal side of the wound was removed and placed in a tube with 1 ml of PBS. The tissue was macerated for 90 s at 5,000 rpm (five cycles), followed by centrifugation (5 min at 2500 RPM). The tissue lysates were 10-folds diluted in PBS and 5 µL were plated on Salted Mannitol Agar. The plates were incubated at 37°C and bacterial load was determined after 24 h. The results were expressed as CFU/g of tissue.

### Dosage of inflammatory mediators and growth factors

The cytokine levels were quantified in the tissue lysates samples using the BD Cytometric Bead Array (CBA) Mouse Th1/Th2/Th17 Cytokine Kit (BD Biosciences, Sao Paulo, Brazil) for detection of IL-2, IL-4, IL-6, IL-10, IL-17A, IFN-γ and TNF-α. The assay was performed in a BD Accuri C6 flow cytometer, following the manufacturer instructions. The Data was analyzed in CBA FCAP Array software (BD Biosciences, Sao Paulo, Brazil). The results were expressed as pg/g of tissue.

The protein concentration in wound tissue was determined using a standard curve of bovine serum albumin (31.25 μg/mL to 500 μg/mL). The amount of nitric oxide (NO) was determined using Griess Reagent and the absorbance values were obtained using a spectrophotometer (Plate reader MB-580; Heales, Shenzhen, China). The results were expressed as Nitrite (μM)/mg of protein. The levels of Vascular Endothelial Growth Factor (VEGF) at wound tissue were measured by the Mouse VEGF ELISA Kit (Sigma-Aldrich; São Paulo, SP, Brazil), following the manufacturer instructions. The results were expressed as pg/mg of protein.

### Statistical analysis

The statistical analysis was conducted in the Graphpad Prism 5.0 (GraphPad Software Inc., San Diego, CA, United States). The data were expressed as mean ± standard error and analyzed by means of One-way Analysis of variance (ANOVA), followed by the Boferroni test. The level of significance was set at 0.05. Area under curve (AUC) was also calculated using Graphpad Prism 8.4.3. The data used in statistical analysis are provided as Supplementary Table S1.

## Results

### *E. brejoensis* essential oil promoted the contraction of *Staphylococcus aureus-*infected wounds

Prior the *in vivo* application, the antimicrobial action of EbEO was confirmed against *S. aureus* ATCC 6538 and a minimum inhibitory concentration (MIC) of 256 μg/mL was obtained as previous reported (Bezerra Filho et al., 2020). The experimental model applied in this work constitutes a local infection of skin wounds. It was observed that the animals recovered well after anesthesia, skin excision and induction of *S. aureus* infection. The weight and temperature of each mouse were analyzed throughout the experiment. All experimental groups had a slight reduction in weight due to the trauma of the lesion and the development of skin infection. In the other days they managed to recover and stabilize their average body weight (data not showed).

**FIGURE 1:**
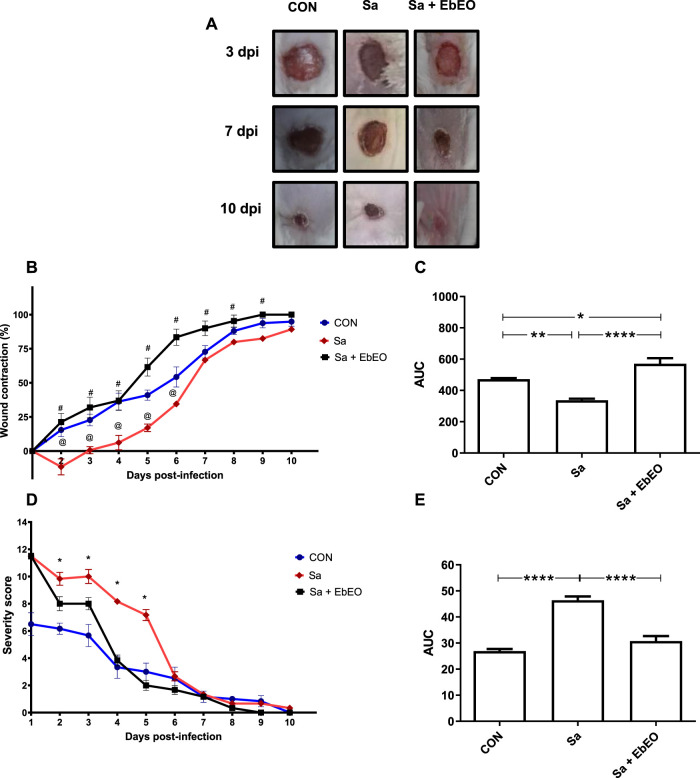
Effects of topical treatment with *Eugenia brejoensis* essential oil on skin lesion contaminated by *Staphylococcus aureus*. **(A)** Representative images of wounds contraction evolution; **(B)** Relative wound contraction values obtained during the 10 days of analysis; **(C)** Area under curve (AUC) of data obtained from wound contraction; **(D)** Analysis of the clinical parameters of the experimental groups during the clinical evaluation period. **(E)** Analysis of the area under the curve (AUC) of the clinical parameters of the mice. (@) Statistical differences of PBS-treated infected wound area (Sa group) and other experimental groups (CON and Sa + Cramoll groups) (*p* < 0.001); (#) Statistical differences of PBS-treated infected wound area (Sa group) and Cramoll-treated infected wounds (Sa + Cramoll group) (*p* < 0.01). *p < 0.05; **p < 0.01; ***p < 0.001; ***p < 0.0001.

*S. aureus* infected wounds showed the lowest wound contraction rates, when compared to other experimental groups (Figures 1A,B). Specifically, *S. aureus* significantly delayed the healing process until 6th dpi (*p* < 0.05), in relation to the uninfected group. In this period, the rates of lesion contraction were 54.28 ± 5.57% and 34.5 ± 2.67% for CON and Sa groups, respectively. The group treated with the EbEO showed faster contraction of *S. aureus*-infected wound with an average contraction of 83.48 ± 11.27% of the lesion area until 6th dpi (*p* < 0.05). EbEO treatment accelerated the healing process even in relation to those animals without infection (*p* < 0.05). This fact is confirmed by the analysis of the AUC from the wound contraction data, where the group treated with EbEO showed the highest AUC values (Figure 1C).

**FIGURE 2:**
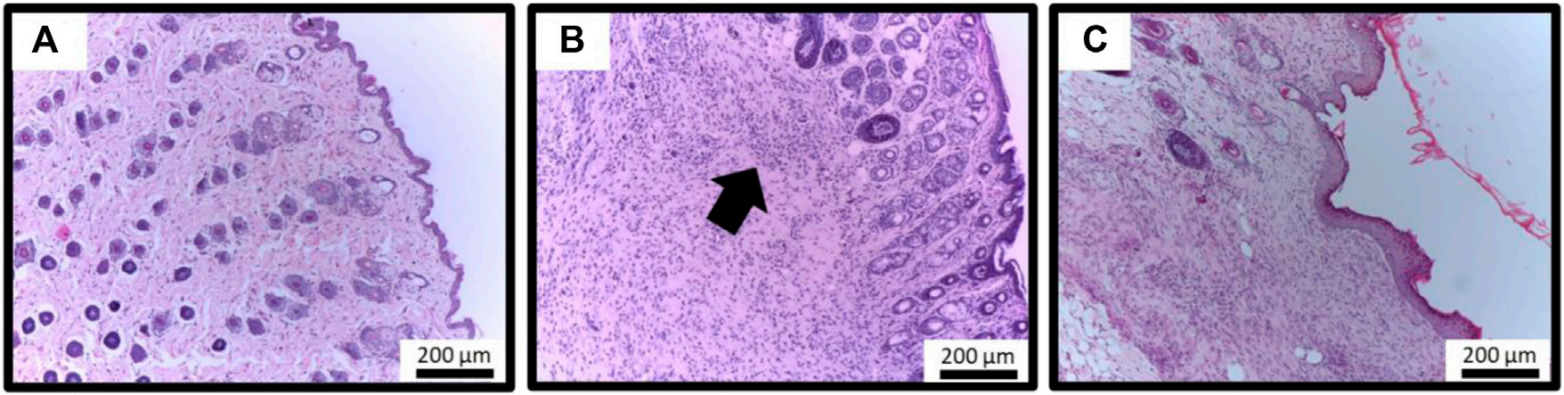
Histological evaluation of topical treatment with EbEO. **(A)** Analysis of wound tissues without *S. aureus* inoculation (CON group); **(B)** Analysis of wound tissues infected by *S. aureus* and without treatment (Sa group); **(C)** Analysis of wound tissues infected by *S. aureus* and treated by EbEO (Sa + EbEO group). The intense proinflammatory infiltrated are highlighted by arrow. 40 X.

The analysis of the score based on clinical parameters showed that presence of *S. aureus* trigged a more severe inflammatory condition until the 5th dpi (Figure 2). In contrast, it was seen that the group treated with EbEO significantly decreased the inflammatory process between the 2^nd^ and 5^th^ dpi (*p* < 0.05), when compared with the infected group (Figure 1D). These data were also confirmed using AUC analysis (Figure 1E).

All these results are confirmed by the histological evaluations (Figure 2), where the PBS-treated wounds infected by *S. aureus* exhibited intense pro-inflammatory infiltrated even after 10 days (Figure 2B). On the other hand, EbEO treatment reduced the presence of immune cells (Figure 2C) and the tissue showed well-evidenced re-epithelialization with moderate cellularity (fibroblasts). In addition, EbEO-treated wounds showed dermis wide vascularization, uniform distribution of collagen fibers and absence of dermal attachments.

### Topical treatment with EbEO reduces the bacterial load in wounds contaminated by *S. aureus*

**FIGURE 3:**
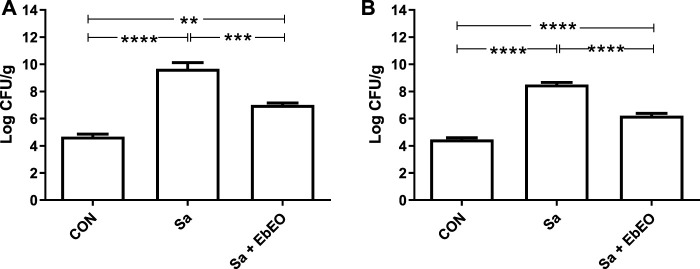
Effect of topical treatment with *Eugenia brejoensis* essential oil on bacterial load in the tissue of wounds contaminated by *Staphylococcus aureus*. **(A)** Bacterial load after 3 days of treatment; **(B)** Bacterial load after 10 days of treatment. **p < 0.05; ***p < 0.001; ****p < 0.0001.

The *in vivo* antimicrobial action of EbEO was confirmed by the quantification of the bacterial load in skin tissue after biopsy at 3rd dpi (Figure 3A) and 10th dpi (Figure 3B). The Sa group presented 9.66 ± 0.71 Log CFU/g and 8.51 ± 0.26 Log CFU/g at 3rd dpi and 10th dpi, respectively. The topical treatment with EbEO significantly reduced (*p* < 0.05) the values of CFU/g in around 30% in both analyzed periods (3rd dpi: 7.0 ± 0.21 Log CFU/; 10th dpi: 6.21 ± 0.23 Log CFU/g).

### EbEO showed potent anti-inflammatory action in wounds contaminated by *S. aureus*

**FIGURE 4:**
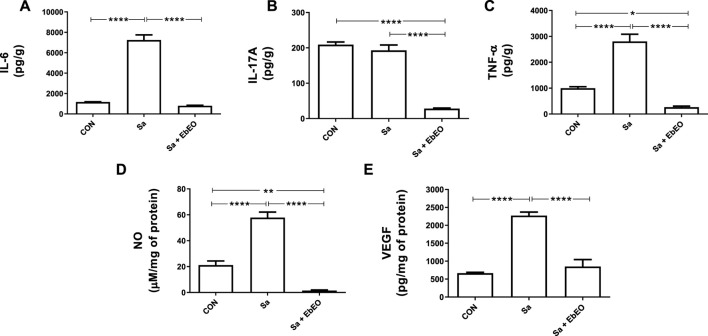
Effect of topical treatment with *Eugenia brejoensis* essential oil on inflammatory markers present in the tissue of wounds contaminated by *Staphylococcus aureus*. **(A)** Amount of IL-6 at wound tissue; **(B)** Amount of IL-17A at wound tissue; **(C)** Amount of TNF-α at wound tissue; **(D)** Amount of NO at wound tissue; **(E)** Amount of VEGF at wound tissue. *p < 0.05; **p < 0.01; ****p < 0.0001.

The levels of tissue cytokines were analyzed at the 3rd dpi and 10th dpi; however, significant results were only observed for IL-6, IL-17A and TNF-α at 3rd dpi (Figure 4). Corroborating with the clinical analysis, the Sa group presented the highest levels (*p* < 0.05) of IL-6 and TNF-α (IL-6: 7243.38 ± 12.05 pg/g of tissue; TNF-α: 2804.67 ± 540.13 pg/g) when compared with CON group (IL-6: 1170.91 ± 42.53 pg/g; TNF-α: 996.78 ± 92.06 pg/g). The IL-17A levels were similar to Sa (192.93 ± 23.23 pg/g) and CON groups (209.0 ± 12.05 pg/g).

The treatment with EbEO significantly decreased (around 90%; *p* < 0.05) the production of IL-6 (803.7 ± 70.27 pg/g), IL-17A (28.13 ± 3.24 pg/g) and TNF-α (266.28 ± 65.52 pg/g) in relation to Sa group. Interestingly, EbEO significantly reduced the levels of IL-17A and TNF-α in relation to CON group (86.54% and 73.29%, respectively; *p* < 0.05).

The measurement of NO and VEGF was performed at 3rd dpi. The wounds from SA group showed higher production of NO (57.86 ± 6.79 μM/mg of protein) and VEGF (2270 ± 196.63 pg/mg of protein) than uninfected wounds (21.14 ± 5.41 μM/mg of protein and 665.37 ± 44.41 pg/mg of protein, respectively). On the other hand, EbEO significantly decreased the local production of these inflammatory mediators (1.50 ± 0.8216 μM/mg of protein and 850.16 ± 270 pg/mg of protein, respectively; *p* < 0.05). It is noteworthy that the treatment with EbEO in the wounds managed to leave the NO values significantly lower than Sa and CON groups (*p* < 0.05).

## Discussion

The skin infectious process is considered one of the factors for the development of bacteremia induced by *S. aureus*, which can invade the bloodstream and spread through the body (Kwiecinski and Horswill, 2020; Macedo et al., 2021). In this study we reported the antimicrobial and anti-inflammatory effects of EbEO in a model of skin wound infection induced by *S. aureus*. Few natural products derived from *Eugenia* plants have been reported as healing agents for skin wounds using *in vitro* or *in vivo* models (Albuquerque et al., 2016; Silva et al., 2018), however the evaluation of these products in bacterial-infected wounds has not been reported. Our results demonstrated that the topical administration of EbEO accelerated the contraction of *S. aureus-*infected wounds by reducing the bacterial load and the levels of inflammatory mediators.

The antimicrobial efficacy of EbEO against *S. aureus* was previously demonstrated using both *in vitro* assays and invertebrate models of infection (*C. elegans* and *G. mellonella*). EbEO was able to inhibit the production of important virulence factors of *S. aureus* such as hemolysin and staphyloxanthin (Bezerra Filho et al., 2020). Thus, the results found in the murine model corroborate those obtained in alternative infection models.

Skin infections caused by *S. aureus* are related to the exacerbated production of pro-inflammatory cytokines as a tumor necrosis factor-α (TNF-α) and IL-6 (Carneiro et al., 2021; Kobayashi et al., 2015). Although essential for the healing and host defense, the excess of inflammatory mediators may result in tissue damage. We observed that EbEO-treated wounds showed decreased severity score than untreated infected lesions, these effects were related with the EbEO antimicrobial action and its anti-inflammatory potential.

Some EO obtained from *Eugenia* plants and EO with similar chemical composition of EbEO have shown anti-inflammatory and antimicrobial effects (Costa et al., 2020a). Moreover, the healing action of β-caryophyllene was shown in a model of skin wound excision in rats where the treatment with this compound decreased the levels of pro-inflammatory cytokines (TNF-α, IFN-γ, IL-1β and IL-6) (Gushiken et al., 2022). In this context, these data are in accordance with the potent anti-inflammatory effects observed for EbEO treatment leading to decreasing levels of NO, VEGF, IL-6, TNF-α and IL-17A, mitigating the intense pro-inflammatory response induced by *S. aureus*.

## Conclusion

In summary, the present study demonstrated that topical treatment with *E. brejoensis* essential oil accelerated the healing process and decreased the severity of *S. aureus-*induced wounds by reducing bacterial load and inflammatory mediators (TNF-α, IL-6, IL-17A, VEGF and NO). These data confirm the anti-infective potential of EbEO and provided new insights into its anti-inflammatory and healing properties, making this oil an interesting alternative for drug development. The cellular and molecular mechanisms involved with EbEO treatment should be better elucidated.

## Data Availability

The raw data supporting the conclusions of this article will be made available by the authors, without undue reservation.
