# Supplementary figures and images for: Antimicrobial and anti-inflammatory effects of Eugenia brejoensis essential oil in mice wounds infected by Staphylococcus aureus
Source: Front Pharmacol. 2022 Oct 14;13:999131. doi: 10.3389/fphar.2022.999131 (PMC9613942; doi:10.3389/fphar.2022.999131)

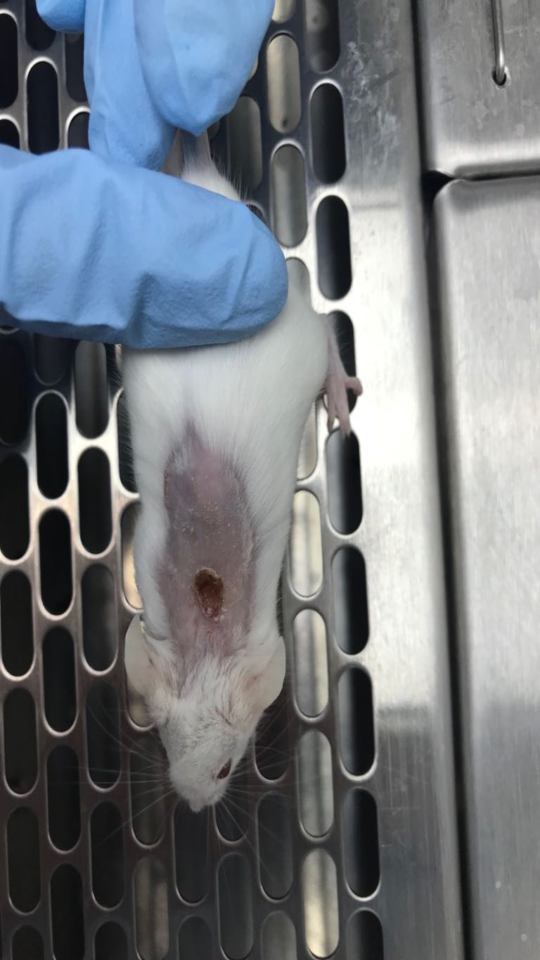

Supplement: Supplementary file 1 [file Image6.TIF]

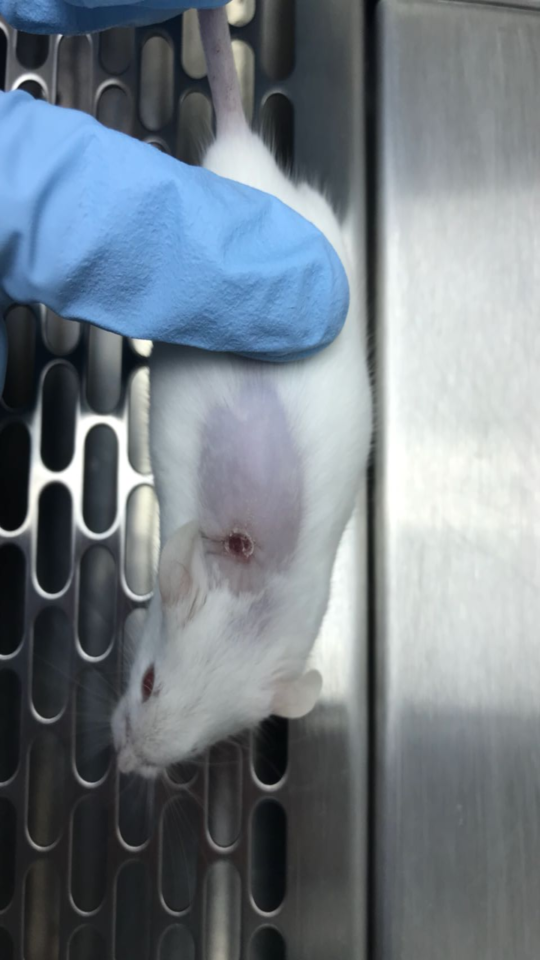

Supplement: Supplementary file 2 [file Image3.TIF]

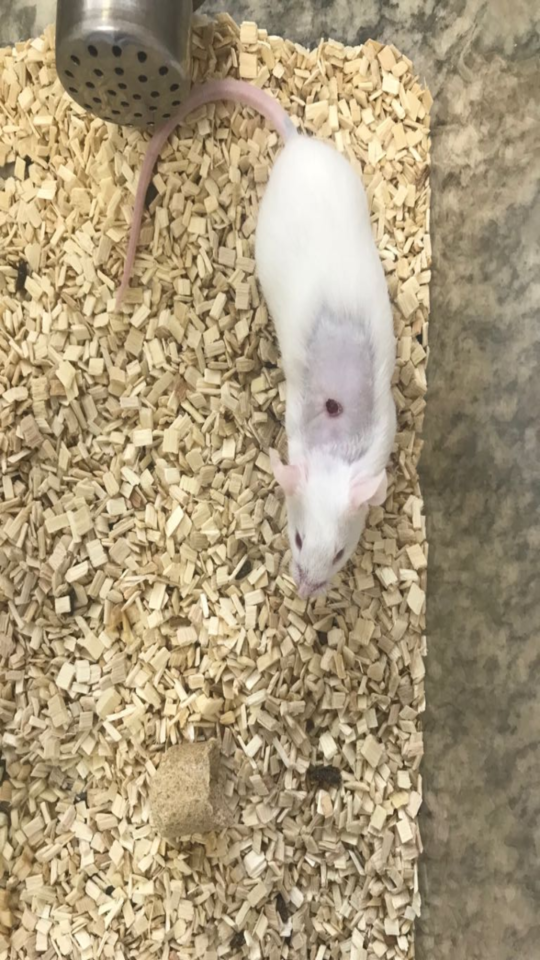

Supplement: Supplementary file 3 [file Image9.TIF]

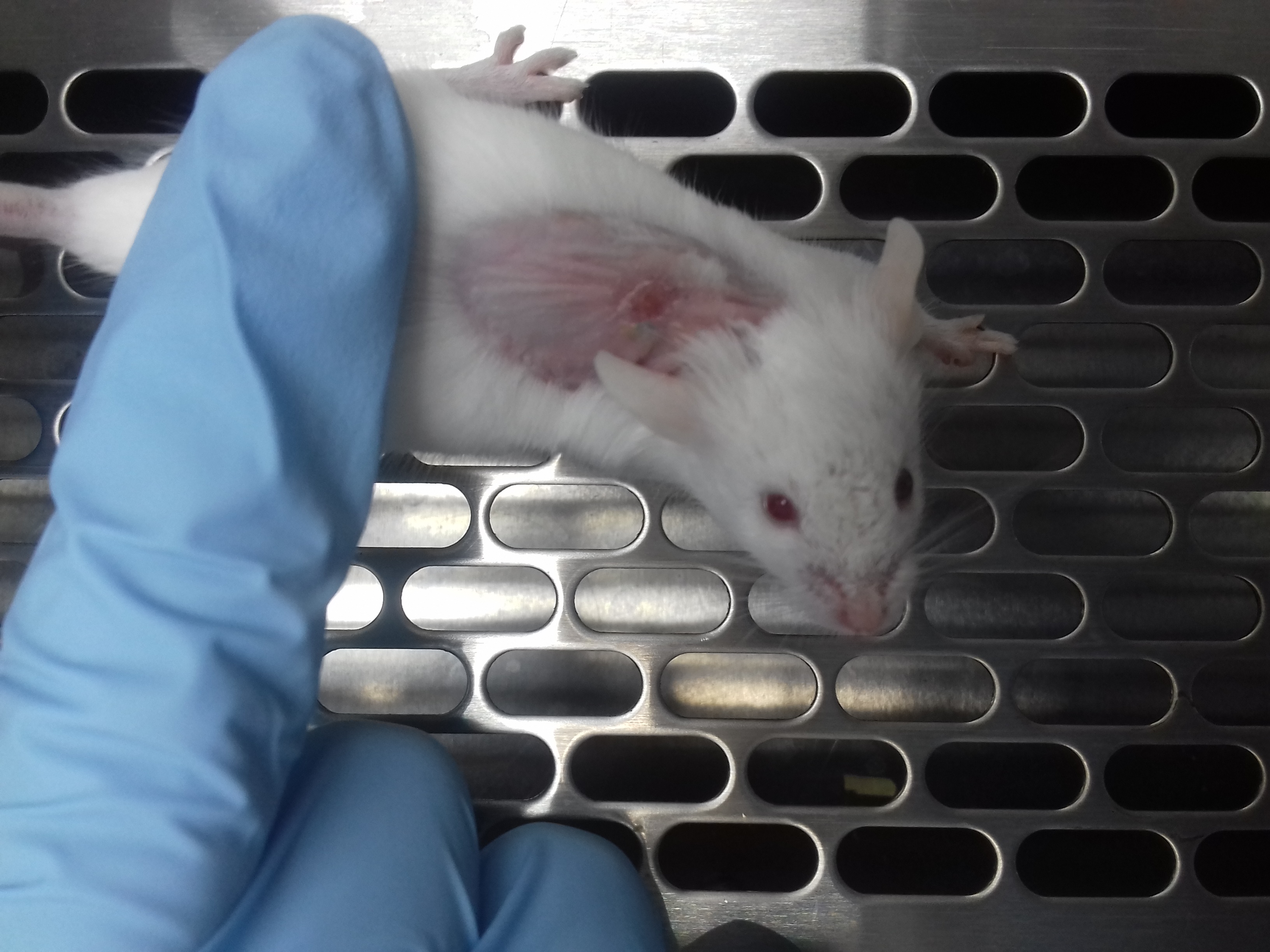

Supplement: Supplementary file 4 [file Image4.JPEG]

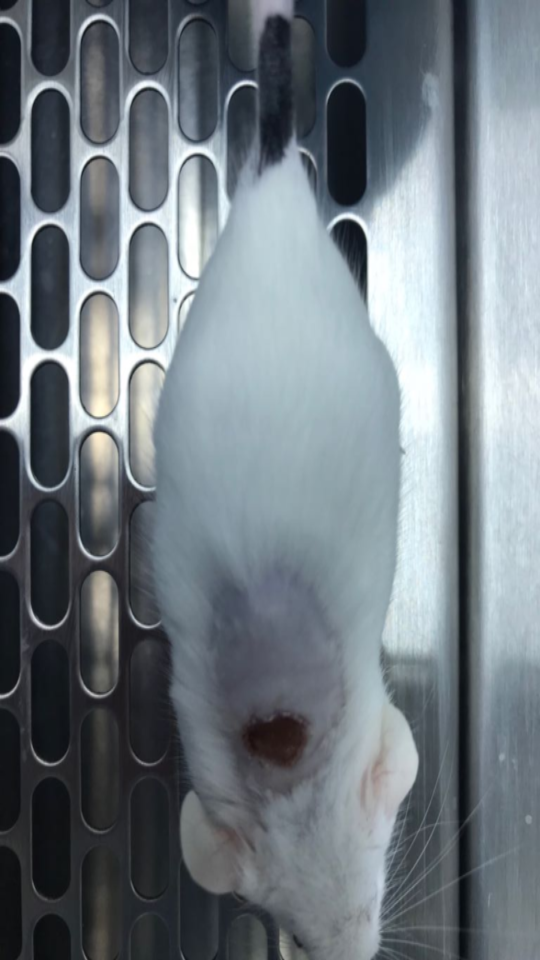

Supplement: Supplementary file 5 [file Image2.TIF]

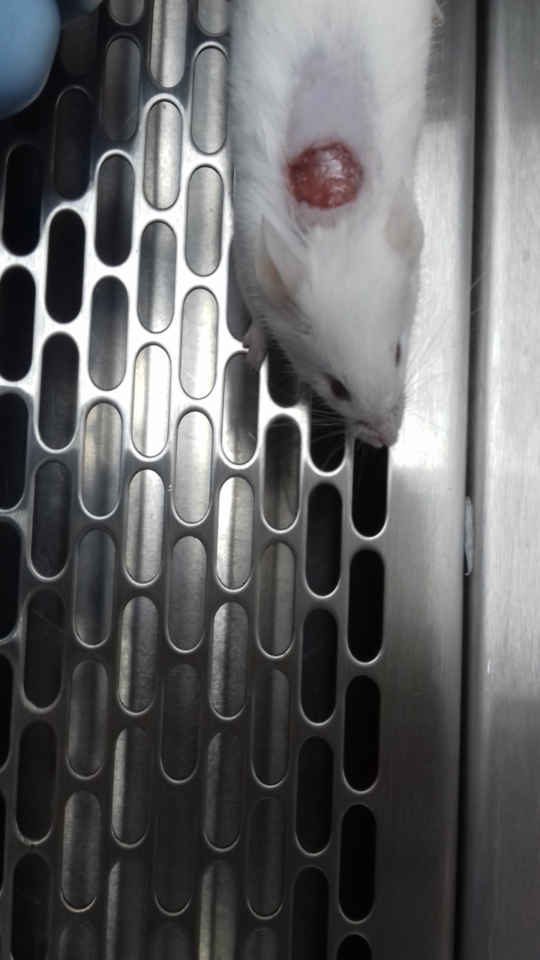

Supplement: Supplementary file 6 [file Image1.TIF]

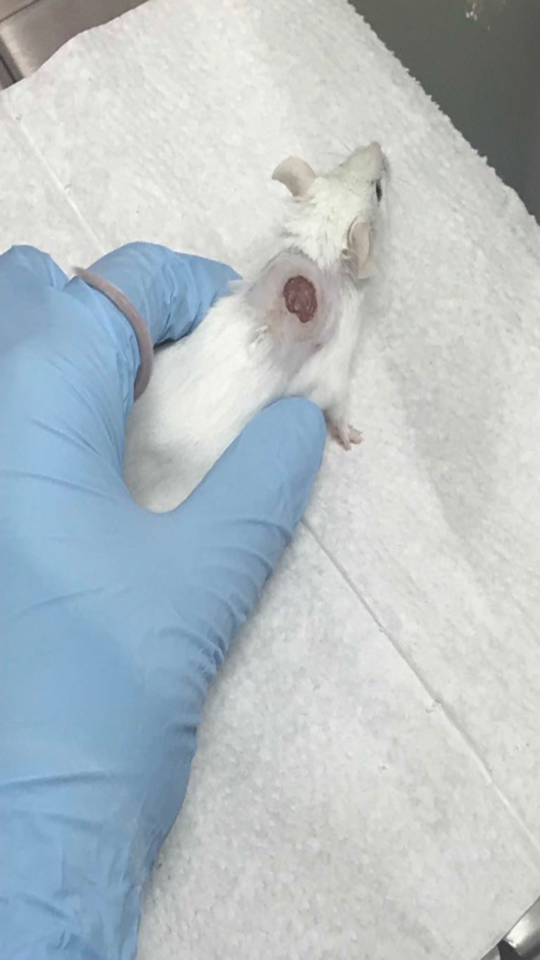

Supplement: Supplementary file 7 [file Image7.TIF]

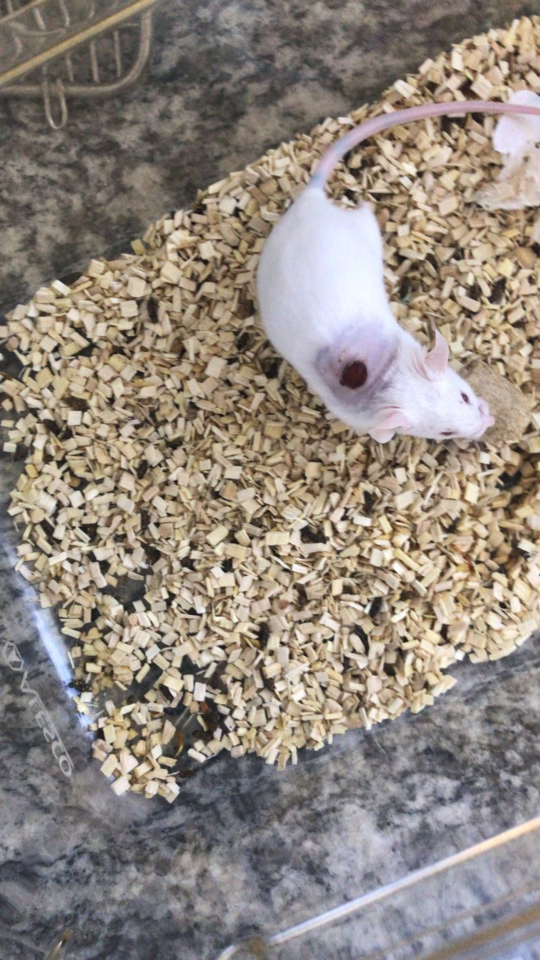

Supplement: Supplementary file 8 [file Image8.TIF]

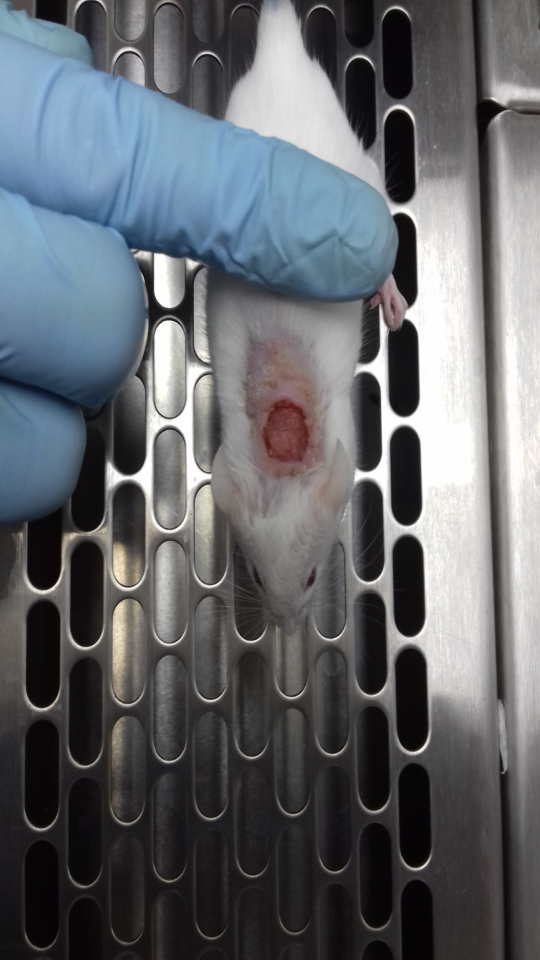

Supplement: Supplementary file 9 [file Image5.TIF]
